# Gallic Acid Enhances Carboplatin-Induced Antitumoral Responses in Cervical Cancer Cells Through Oxidative Stress-Associated Mitochondrial and Apoptotic Mechanisms

**DOI:** 10.3390/biomedicines14061399

**Published:** 2026-06-21

**Authors:** Mehmet Emin Ayağ, Mehmet Cudi Tuncer, İlhan Özdemir

**Affiliations:** 1 Mehmet Emin Ayağ Practice, Department of Gynecology and Obstetrics, 47000 Mardin, Turkey; drmehmetemin.gyn@hotmail.com; 2 Department of Anatomy, Faculty of Medicine, Dicle University, 21280 Diyarbakır, Turkey; 3 Department of Histology and Embryology, Faculty of Medicine, Kahramanmaraş Sütçü İmam University, 46000 Kahramanmaraş, Turkey; ilhanozdemir32@hotmail.com

**Keywords:** gallic acid, carboplatin, HeLa, HaCaT, apoptosis, reactive oxygen species

## Abstract

**Background/Objectives:** Gallic acid (GA) is a naturally occurring polyphenol with reported antioxidant and anticancer properties. This study investigated whether GA enhances carboplatin (CARB)-associated anticancer activity in HeLa cervical cancer cells through mechanisms related to oxidative stress, mitochondrial dysfunction, apoptosis, and cell cycle dysregulation, while comparatively evaluating cytotoxicity in HaCaT cells. **Methods:** The effects of GA and CARB, individually and in combination, were evaluated using cell viability assays, apoptosis and cell cycle analyses, intracellular reactive oxygen species (ROS) measurements, N-acetylcysteine (NAC)-mediated rescue experiments, mitochondrial membrane potential assessment, reverse transcription–quantitative polymerase chain reaction (RT-qPCR), immunocytochemistry, and three-dimensional (3D) tumor spheroid models. Bioinformatic analyses were performed to explore pathways associated with the observed molecular responses. **Results:** The GA + CARB combination demonstrated enhanced cytotoxicity and apoptotic activity in HeLa cells compared with either monotherapy, while exhibiting comparatively lower toxicity in HaCaT cells. Combination treatment increased intracellular ROS levels, whereas NAC pretreatment partially reversed ROS accumulation and cytotoxicity, supporting a contributory role of oxidative stress in treatment-associated responses. The combination also induced mitochondrial membrane depolarization, increased G2/M arrest and SubG1 accumulation, and modulated apoptosis- and cell cycle-related gene expression. In 3D spheroid models, GA + CARB reduced spheroid growth and viability and disrupted spheroid integrity more effectively than single-agent treatments. Bioinformatic analyses identified interconnected pathways associated with oxidative stress, apoptosis, and cell cycle regulation. **Conclusions:** GA may enhance CARB-associated anticancer activity through mechanisms linked to oxidative stress, mitochondrial dysfunction, apoptosis, and cell cycle dysregulation. The incorporation of ROS/NAC rescue experiments and 3D spheroid validation further supports the biological relevance of the observed effects. Nevertheless, these findings remain preliminary and require confirmation in advanced in vivo and translational cervical cancer models.

## 1. Introduction

Despite major advances in prevention, diagnosis, and therapeutic intervention, cancer continues to represent a substantial global health burden and remains one of the leading causes of mortality worldwide [1]. Among gynecological malignancies, cervical cancer constitutes a major public health concern, particularly in low- and middle-income countries where screening programs and vaccination coverage remain insufficient. Persistent infection with high-risk Human Papillomavirus (HPV) subtypes is recognized as the principal etiological factor underlying cervical carcinogenesis [2]. Although multimodal therapeutic approaches involving surgery, radiotherapy, and chemotherapy have improved clinical outcomes in selected patient groups, treatment-associated toxicity, tumor recurrence, and the development of chemoresistance continue to limit long-term therapeutic success [3].

Platinum-based compounds remain integral components of systemic treatment protocols for various solid malignancies. CARB, a second-generation platinum analog developed to reduce the severe toxicity profile associated with cisplatin, exerts its cytotoxic activity primarily through the formation of intra- and inter-strand DNA cross-links, ultimately impairing DNA replication and promoting apoptotic cell death [4]. Nevertheless, the clinical utility of CARB is frequently compromised by cumulative adverse effects, including hematological toxicity, nephrotoxicity, and the gradual emergence of resistance-associated cellular adaptations [5]. These limitations have intensified interest in combination-based therapeutic strategies capable of enhancing anticancer efficacy while potentially reducing the required chemotherapeutic dose burden.

Naturally derived bioactive compounds have gained considerable attention as potential adjuvant agents in cancer therapy because of their pleiotropic biological activities and relatively favorable safety profiles [6]. GA (GA; 3,4,5-trihydroxybenzoic acid) is a phenolic compound broadly distributed in fruits, medicinal plants, tea, and various dietary sources [7]. Increasing evidence indicates that GA exhibits anticancer activity through multiple interconnected mechanisms, including modulation of intracellular oxidative balance, mitochondrial dysfunction, induction of apoptosis, and regulation of cell cycle progression [8,9]. Although GA has traditionally been recognized for its antioxidant properties, accumulating data suggest that under specific intracellular conditions, particularly in malignant cells characterized by elevated basal oxidative stress, GA may exert a pro-oxidant effect capable of selectively promoting cancer cell death [10]. This context-dependent redox-modulating capacity may provide a therapeutic advantage by increasing the susceptibility of tumor cells to oxidative injury while relatively sparing non-malignant cells.

ROS play a central role in the regulation of cancer cell survival and death. Excessive ROS accumulation can disrupt mitochondrial homeostasis, resulting in mitochondrial membrane depolarization, cytochrome c release, caspase cascade activation, and initiation of intrinsic apoptotic signaling pathways [11]. In parallel, oxidative stress has also been associated with DNA damage-associated checkpoint activation and dysregulation of cell cycle progression, both of which contribute to the suppression of malignant cell proliferation. Functional antioxidant rescue experiments using NAC are widely utilized to determine whether treatment-associated cytotoxicity is mechanistically linked to ROS-mediated pathways [12]. Such approaches provide important mechanistic insight into the contribution of oxidative stress to apoptosis and therapeutic responsiveness.

The HeLa cell line, derived from HPV-18-positive cervical adenocarcinoma, represents one of the most extensively characterized in vitro cervical cancer models and is widely employed in mechanistic anticancer research [13]. In contrast, HaCaT cells are immortalized human keratinocytes commonly used as a comparative nontumoral epithelial model in cytotoxicity studies [14]. Simultaneous evaluation of these two cell lines enables comparative assessment of treatment selectivity and provides preliminary insight into differential responses between malignant and non-malignant cellular systems.

Combination-based chemotherapy strategies offer several potential advantages compared with monotherapy, including synergistic anticancer activity, reduced effective drug concentrations, and improved capacity to overcome resistance-associated mechanisms [15]. In our recent study, we demonstrated that GA enhanced cisplatin-associated anticancer activity in HeLa cervical cancer cells through coordinated modulation of apoptosis, cell cycle progression, and stress-associated signaling pathways [16]. Although these findings supported the therapeutic potential of GA as an adjuvant to platinum-based chemotherapy, cisplatin-associated toxicity remains a major clinical limitation despite its established anticancer efficacy. By contrast, carboplatin exhibits a more favorable tolerability profile while retaining the therapeutic advantages of platinum-based treatment. Therefore, determining whether GA can similarly potentiate carboplatin-associated anticancer responses represents an important extension of our previous findings and may provide additional translational relevance for platinum-based combination therapy. In particular, integrated evaluations addressing oxidative stress, mitochondrial dysfunction, apoptosis, cell cycle regulation, and treatment responsiveness under physiologically relevant three-dimensional (3D) tumor-like conditions remain limited. Moreover, the extent to which ROS-associated mechanisms contribute functionally to the cytotoxic activity of the GA + CARB combination has not been comprehensively investigated using antioxidant rescue approaches in both conventional monolayer and 3D spheroid systems.

Accordingly, the present study was designed to investigate whether GA enhances CARB-associated anticancer activity in cervical cancer cells through interconnected mechanisms involving oxidative stress, mitochondrial dysfunction, apoptotic signaling, and disruption of cell cycle progression. To address this objective, the effects of GA and CARB, individually and in combination, were evaluated in HeLa cervical carcinoma cells and comparatively assessed in HaCaT immortalized keratinocytes. Multiple complementary experimental approaches were employed, including cell viability analyses, flow cytometry-based apoptosis and cell cycle assessments, intracellular ROS quantification, NAC-mediated antioxidant rescue experiments, ΔΨm analysis, RT-qPCR-based gene expression profiling, immunocytochemical evaluation, and bioinformatic pathway analyses. Furthermore, three-dimensional (3D) tumor spheroid experiments were performed to investigate treatment-associated effects on spheroid growth dynamics, structural integrity, viability, live/dead cell distribution, and ROS-related cytotoxicity under tumor-mimicking conditions. Through this integrative experimental framework, this study aimed to provide a more comprehensive mechanistic characterization of the GA + CARB combination and to evaluate its potential relevance as a natural compound-supported therapeutic strategy for cervical cancer.

## 2. Materials and Methods

### 2.1. Reagents, Stock Solution Preparation, and Treatment Conditions

GA (GA; ≥97% purity; CAS No. 149-91-7), CARB (CARB; ≥98% purity), dimethyl sulfoxide (DMSO), and NAC were obtained from Sigma-Aldrich (St. Louis, MO, USA). GA was initially dissolved in DMSO to prepare a 100 mM stock solution and subsequently diluted with complete culture medium to obtain working concentrations of 10, 20, 50, 100, 250, and 500 µM. CARB was dissolved in sterile distilled water to generate a 10 mM stock solution and further diluted in culture medium to final concentrations of 1, 2.5, 5, 10, 25, and 50 µM. In all experimental groups, the final DMSO concentration was maintained below 0.1% (*v*/*v*) to minimize solvent-associated cytotoxic effects.

To examine the functional involvement of oxidative stress in the biological responses induced by the GA + CARB combination, cells were pre-incubated with N-acetylcysteine (NAC) before treatment. A 100 mM NAC stock solution was prepared in phosphate-buffered saline (PBS), and cells were exposed to NAC at a working concentration of 5 mM for 1 h. The antioxidant intervention was implemented in both two-dimensional (2D) monolayer cultures and three-dimensional (3D) tumor spheroid models, allowing for assessment of the extent to which reactive oxygen species (ROS)-dependent processes contributed to the observed cytotoxic effects.

### 2.2. Cell Culture Conditions

HeLa human cervical adenocarcinoma cells (ATCC^®^ CCL-2™; American Type Culture Collection, Manassas, VA, USA) and HaCaT immortalized human keratinocytes (Cell Lines Service, CLS; Cat. No. 300493, Eppelheim, Germany) were used in this study. Both cell lines were maintained in Dulbecco’s Modified Eagle Medium (DMEM; Gibco, Thermo Fisher Scientific, Waltham, MA, USA) supplemented with 10% heat-inactivated fetal bovine serum (FBS; Gibco, Thermo Fisher Scientific), 100 U/mL penicillin, and 100 µg/mL streptomycin (Gibco, Thermo Fisher Scientific).

Cell cultures were maintained at 37 °C under humidified conditions with 5% CO_2_. Fresh culture medium was supplied at intervals of 2–3 days, and cells were passaged when approximately 80% confluence was reached using a 0.25% trypsin–EDTA solution (Gibco, Thermo Fisher Scientific). To reduce the potential influence of passage-related phenotypic alterations, all experiments were conducted using cells between passages 5 and 15.

### 2.3. Cell Viability and Combination Treatment Analysis

Cell viability following exposure to GA, CARB, and their combined treatment was determined using the 3-(4,5-dimethylthiazol-2-yl)-2,5-diphenyltetrazolium bromide (MTT) assay. For this purpose, HeLa and HaCaT cells were plated in 96-well culture plates at a density of 5 × 10^3^ cells per well and allowed to adhere for 24 h. After the stabilization period, cells were treated with GA (10–500 µM) or CARB (1–50 µM), either as single agents or in combination, and maintained under standard culture conditions for 24 or 48 h prior to viability assessment.

At the end of the treatment period, culture media were carefully removed, and cells were incubated with MTT solution prepared in phosphate-buffered saline (PBS) at a final concentration of 5 mg/mL (Sigma-Aldrich, St. Louis, MO, USA). After 4 h at 37 °C, the purple formazan product generated by metabolically active cells was dissolved in 150 µL of DMSO. Optical density was then recorded using a microplate reader at 570 nm with 630 nm as the reference wavelength. Viability values were expressed as percentages relative to the untreated control group.

Dose–response curves were fitted by nonlinear regression using a sigmoidal model, and the corresponding half-maximal inhibitory concentration (IC_50_) values were calculated with GraphPad Prism version 9.0 (GraphPad Software Inc., San Diego, CA, USA). IC_50_ values were subsequently used as operational reference concentrations for combination treatment experiments. Drug interaction profiles were evaluated according to the Chou–Talalay method using CompuSyn software (ComboSyn Inc., Paramus, NJ, USA). CI values were interpreted as follows: CI < 1 indicated synergistic interaction, CI = 1 indicated additive interaction, and CI > 1 indicated antagonistic interaction.

### 2.4. Flow Cytometric Evaluation of Apoptosis

Flow cytometric assessment of cell death was performed using Annexin V-fluorescein isothiocyanate (FITC) and propidium iodide (PI) dual staining to distinguish apoptotic and necrotic populations. For these experiments, HeLa and HaCaT cells were plated in 6-well plates at a density of 2 × 10^5^ cells per well and maintained overnight to ensure adequate attachment. The cultures were then exposed to GA, CARB, or their combination at the established treatment concentrations for 48 h prior to analysis.

To ensure accurate quantification of treatment-induced cell death, both attached and detached cells were recovered after the incubation period and pooled for analysis. The collected cells were washed twice with ice-cold phosphate-buffered saline (PBS) before staining with the Annexin V-FITC/PI Apoptosis Detection Kit (BD Biosciences, San Jose, CA, USA) following the supplier’s protocol. For staining, cell pellets were suspended in 1× binding buffer and incubated with 5 µL Annexin V-FITC together with 5 µL propidium iodide (PI) for 15 min at room temperature under light-protected conditions.

Flow cytometric data acquisition was performed immediately after staining using a BD FACSCanto™ II instrument (BD Biosciences, San Jose, CA, USA). A minimum of 20,000 events was acquired for each sample. Debris exclusion and population gating were initially performed according to FSC/SSC distribution characteristics prior to fluorescence-based quadrant analysis. Data analysis was performed using FlowJo software version 10.8 (BD Biosciences). Cell populations were classified as viable (Annexin V^−^/PI^−^), early apoptotic (Annexin V^+^/PI^−^), late apoptotic (Annexin V^+^/PI^+^), or necrotic (Annexin V^−^/PI^+^) based on quadrant distribution profiles.

### 2.5. Determination of Intracellular ROS and NAC-Mediated Antioxidant Rescue

Changes in intracellular reactive oxygen species (ROS) levels were monitored using the oxidation-sensitive fluorescent dye 2′,7′-dichlorodihydrofluorescein diacetate (DCFH-DA; Sigma-Aldrich, St. Louis, MO, USA). For these experiments, HeLa and HaCaT cells were cultured in 6-well plates and maintained in the presence of GA, CARB, or their combined treatment at the established experimental concentrations for 48 h before ROS assessment.

Following treatment, cells were incubated with 10 µM DCFH-DA at 37 °C for 30 min in the dark. After staining, cells were harvested by trypsinization, washed twice with ice-cold PBS, and immediately analyzed by flow cytometry using a BD FACSCanto™ II instrument (BD Biosciences, San Jose, CA, USA). Fluorescence signals were detected using 488 nm excitation and 525 nm emission settings. Intracellular ROS levels were expressed as mean fluorescence intensity (MFI) normalized to untreated control groups. Data acquisition and analysis were performed using FlowJo software version 10.8 (BD Biosciences).

To assess whether oxidative stress contributed to the cellular responses induced by the GA + CARB combination, N-acetylcysteine (NAC) was employed as an antioxidant intervention. Before treatment, cultures were incubated with 5 mM NAC for 1 h and subsequently exposed to the GA + CARB regimen. The effects of NAC on intracellular reactive oxygen species (ROS) levels and cellular responses to treatment were further examined in both 2D monolayer cultures and 3D spheroid models.

### 2.6. Assessment of ΔΨm

Changes in ΔΨm were evaluated using the cationic fluorescent probe JC-1 (5,5′,6,6′-tetrachloro-1,1′,3,3′-tetraethylbenzimidazolocarbocyanine iodide; Cayman Chemical, Ann Arbor, MI, USA). HeLa and HaCaT cells were seeded into 6-well plates and treated with GA, CARB, or the GA + CARB combination for 48 h under standard culture conditions.

Following treatment, cells were incubated with JC-1 dye at a final concentration of 2 µM in serum-free culture medium for 30 min at 37 °C in the dark. After staining, cells were washed twice with PBS to remove excess dye and subsequently analyzed by flow cytometry using a BD FACSCanto™ II flow cytometer (BD Biosciences, San Jose, CA, USA).

Mitochondrial polarization status was determined based on the fluorescence emission shift of JC-1 dye from red fluorescent aggregates to green fluorescent monomers. Red fluorescence corresponding to polarized mitochondria was detected in the PE channel (FL-2), whereas green fluorescence associated with depolarized mitochondria was detected in the FITC channel (FL-1). The ratio of red-to-green fluorescence intensity was calculated as an indicator of mitochondrial membrane integrity. Carbonyl cyanide m-chlorophenyl hydrazone (CCCP; 50 µM for 30 min), a mitochondrial oxidative phosphorylation uncoupler, was used as a positive control for mitochondrial depolarization. Data acquisition and analysis were performed using FlowJo software version 10.8 (BD Biosciences).

### 2.7. Flow Cytometric Analysis of Cell Cycle Distribution

Cell cycle progression was evaluated by PI-based DNA content analysis following treatment with GA, CARB, or the GA + CARB combination. For treatment experiments, HeLa cells were cultured in 6-well plates and allowed to stabilize under standard incubation conditions. The cultures were then treated with GA, CARB, or their combination at concentrations corresponding to the respective IC_50_ values for 48 h, whereas untreated cultures were included as controls.

At the end of the treatment period, cells were harvested by trypsinization and washed twice with ice-cold PBS. Cell pellets were fixed overnight in 70% ethanol at 4 °C to preserve nuclear DNA content. Following fixation, samples were centrifuged, washed with PBS to remove residual ethanol, and incubated with RNase A solution (100 µg/mL) for 30 min at 37 °C in order to eliminate RNA-associated fluorescence interference. Subsequently, cells were stained with PI (PI; 50 µg/mL) for 30 min at room temperature in the dark.

Cell cycle analysis was conducted by measuring DNA content with a BD FACSCanto™ II flow cytometer (BD Biosciences, San Jose, CA, USA). For each experimental condition, at least 10,000 cellular events were acquired. Prior to quantification of cell cycle compartments, gating strategies were applied to remove debris and cell aggregates. The proportions of cells within the SubG1, G0/G1, S, and G2/M phases were subsequently calculated using FlowJo software version 10.8 (BD Biosciences). The SubG1 fraction was interpreted as an indicator of apoptotic DNA fragmentation associated with treatment-induced cell death.

### 2.8. RT-qPCR-Based Gene Expression Profiling

Total RNA was extracted from treated and untreated cells using the RNeasy Mini Kit (Qiagen, Hilden, Germany) in accordance with the manufacturer’s protocol. The quantity and quality of isolated RNA were assessed using a NanoDrop™ 2000 spectrophotometer (Thermo Fisher Scientific, Waltham, MA, USA). Only samples with an A_260_/A_280_ ratio of at least 1.8 were included in subsequent molecular analyses.

Complementary DNA (cDNA) synthesis was carried out using 1 µg of total RNA with the RevertAid First Strand cDNA Synthesis Kit (Thermo Fisher Scientific) according to the manufacturer’s recommendations. Reverse transcription reactions were performed at 42 °C for 60 min, followed by enzyme inactivation under recommended thermal conditions.

Quantitative real-time polymerase chain reaction (RT-qPCR) was performed using SYBR™ Green Master Mix (Applied Biosystems, Foster City, CA, USA) and a StepOnePlus™ Real-Time PCR System (Applied Biosystems). Thermal cycling was initiated with a denaturation step at 95 °C for 10 min, followed by 40 cycles consisting of 95 °C for 15 s and 60 °C for 1 min. Gene-specific primers were selected from published studies and their specificity was verified using the NCBI Primer-BLAST tool. The primer sequences employed for amplification are listed in Table 1.

Gene expression analyses included apoptosis-associated transcripts (*BAX, BCL2, CASP3, CASP9, TP53,* and *CYCS*) together with genes involved in cell cycle regulation (*CDKN1A/p21, CDKN2A/p16, CDK4,* and *CDK6*). For data normalization, *GAPDH* and *ACTB (β-actin)* served as the housekeeping reference genes. Relative transcript levels were determined using the comparative 2^−ΔΔCt^ method. To improve analytical reliability and limit technical variation, each reaction was carried out in triplicate.

### 2.9. Immunocytochemical Evaluation of Active Caspase-3 Expression

Immunocytochemical staining was performed to evaluate treatment-associated changes in active caspase-3 protein expression using a 3,3′-diaminobenzidine (DAB)-based chromogenic detection system. HeLa and HaCaT cells were seeded onto sterile glass coverslips placed in culture plates and exposed to the designated treatment conditions under standard incubation parameters. After completion of the treatment period, cellular fixation was carried out using 4% paraformaldehyde (Sigma-Aldrich, St. Louis, MO, USA) for 15 min at room temperature.

To suppress endogenous peroxidase activity, fixed cells were incubated in methanol containing 3% hydrogen peroxide (H_2_O_2_) for 10 min at room temperature. Membrane permeabilization was subsequently achieved using PBS containing 0.3% Triton™ X-100 (Sigma-Aldrich) for 10 min. To minimize nonspecific antibody binding, samples were blocked with 5% normal goat serum prepared in PBS for 1 h at room temperature.

Primary antibody labeling was performed by maintaining the cells overnight at 4 °C in a humidified chamber containing rabbit anti-active caspase-3 antibody (1:200; Cat. No. ab184787, Abcam, Cambridge, UK). Following PBS washing steps, samples were incubated with biotinylated secondary antibody (anti-rabbit IgG, 1:500 dilution) for 30 min at room temperature. Signal amplification was performed using a streptavidin–horseradish peroxidase (HRP) complex (Vectastain^®^ ABC Elite Kit, Vector Laboratories, Burlingame, CA, USA) according to the manufacturer’s protocol.

Chromogenic visualization was achieved using a DAB substrate kit (Sigma-Aldrich). The development of brown-colored immunoreactivity was monitored microscopically and terminated by rinsing with distilled water once adequate staining intensity was observed. Nuclear counterstaining was performed using Mayer’s hematoxylin (Sigma-Aldrich). Following dehydration through graded ethanol solutions and xylene treatment, coverslips were mounted using Entellan^®^ mounting medium (Merck, Darmstadt, Germany).

Microscopic evaluation of the stained samples was carried out using an Olympus BX53 light microscope (Olympus Corporation, Tokyo, Japan). Semi-quantitative evaluation of active caspase-3 immunoreactivity was performed using the Histochemical Score (H-score) method based on staining intensity and the percentage of positively stained cells.

### 2.10. Three-Dimensional (3D) Tumor Spheroid Formation, Treatment, and Functional Analyses

To evaluate treatment responses in a setting that more closely reflects key features of solid tumors, including multicellular architecture, diffusion gradients, and intercellular interactions, a 3D spheroid model was employed. HeLa cells were cultured in Dulbecco’s Modified Eagle Medium (DMEM; Gibco, Thermo Fisher Scientific, Waltham, MA, USA) supplemented with 10% fetal bovine serum (FBS) and 1% penicillin–streptomycin, and maintained at 37 °C in a humidified incubator containing 5% CO_2_.

To initiate spheroid formation, exponentially growing HeLa cells were dispensed into ultra-low-attachment round-bottom 96-well plates (Corning Inc., Corning, NY, USA). A density of 3 × 10^3^ cells per well was used, with each well containing 200 µL of complete culture medium. Plates were subsequently incubated under standard culture conditions to allow for spontaneous spheroid aggregation and maturation. Compact and morphologically uniform spheroids formed within 48–72 h and were selected for downstream analyses. Only spheroids displaying regular morphology and comparable diameter ranges were included in experimental evaluations to minimize inter-spheroid variability.

Following spheroid formation, culture medium was carefully replaced with fresh medium containing GA, CARB, or the GA + CARB combination. Treatment concentrations were selected according to IC_50_ values obtained from two-dimensional (2D) monolayer experiments and were used as operational reference concentrations for 3D studies. Because multicellular spheroids typically exhibit slower growth rates and may restrict drug diffusion into the spheroid core, treatments were continued for 72 h under standard culture conditions. Control spheroids received the corresponding vehicle treatment. Unless indicated otherwise, all experiments involving spheroid models were conducted using a minimum of three independent biological replicates.

#### 2.10.1. Bright-Field Imaging and Morphological Evaluation of Spheroids

Treatment-related changes in spheroid architecture were documented using an inverted bright-field microscope (Olympus CKX53, Olympus Corporation, Tokyo, Japan). To facilitate reliable comparisons among experimental groups, all images were acquired under standardized optical and exposure conditions. Qualitative evaluation focused on spheroid compactness, preservation of structural organization, border morphology, and evidence of cellular scattering or fragmentation.

#### 2.10.2. Evaluation of Treatment-Associated Changes in Spheroid Diameter

Spheroid size measurements were performed using ImageJ software (version 1.53; National Institutes of Health, Bethesda, MD, USA). For each spheroid, diameter values were calculated as the average of two perpendicular measurements obtained from the widest spheroid regions. Quantitative analyses were conducted using spheroids derived from at least three independent experiments.

#### 2.10.3. ATP-Based Viability Analysis of 3D Tumor Spheroids

Cell viability within spheroids was determined using the CellTiter-Glo^®^ 3D Cell Viability Assay (Promega, Madison, WI, USA), which quantifies intracellular ATP as an indicator of metabolically active cells. Following treatment, an equal volume of CellTiter-Glo^®^ 3D reagent was directly added to each well to facilitate complete spheroid lysis and ATP release. After completion of the manufacturer-specified incubation period required for signal stabilization, luminescence was quantified with a microplate reader (BioTek Instruments, Winooski, VT, USA). The resulting values were normalized against vehicle-treated spheroids and expressed as percentages relative to the control group.

#### 2.10.4. Evaluation of Spheroid Viability by Live/Dead Fluorescence Analysis

Fluorescence-based viability assessment was performed to further characterize treatment-induced cytotoxic effects within spheroids using Calcein-AM and Ethidium homodimer-1 (Thermo Fisher Scientific, Waltham, MA, USA). After completion of the staining procedure, spheroids were imaged with an inverted fluorescence microscope (Olympus CKX53, Olympus Corporation, Tokyo, Japan) equipped with the appropriate excitation and emission filter configurations.

Images were acquired using identical focal planes, exposure parameters, and acquisition settings across all groups without post-acquisition image enhancement or manipulation. Green fluorescence signals corresponding to viable cells and red fluorescence signals corresponding to non-viable cells were quantitatively analyzed using ImageJ software (version 1.53; National Institutes of Health). Quantitative estimates of viable and non-viable cell populations were derived from fluorescence intensity data obtained from a minimum of three independent biological replicates. The resulting fluorescence profiles were analyzed in conjunction with morphological observations to characterize the localization and distribution of living and damaged cells throughout the 3D spheroid architecture.

#### 2.10.5. NAC-Mediated Antioxidant Rescue Experiments in 3D Spheroids

The involvement of oxidative stress in spheroid responses to treatment was investigated through antioxidant intervention with NAC. Fully established HeLa spheroids were incubated with 5 mM NAC (Sigma-Aldrich, St. Louis, MO, USA) for 1 h at 37 °C under humidified conditions containing 5% CO_2_ before administration of GA, CARB, or their combination. This experimental setting was chosen to promote intracellular ROS neutralization while avoiding substantial nonspecific toxicity associated with antioxidant exposure.

Following NAC pretreatment, spheroids were exposed to treatment conditions corresponding to previously determined IC_50_-based concentrations and maintained for 72 h under standard culture conditions. Untreated spheroids and NAC-only groups were included as experimental controls.

#### 2.10.6. Quantification of Intracellular ROS Levels in 3D Spheroids

Assessment of intracellular ROS levels in spheroid-derived cells was performed using DCFH-DA (DCFH-DA; Sigma-Aldrich, St. Louis, MO, USA). At the end of the treatment period, spheroids were converted into single-cell suspensions through enzymatic digestion with Accutase^®^ solution (Sigma-Aldrich) for 15 min at 37 °C. The resulting cells were subsequently washed with PBS and exposed to 10 µM DCFH-DA for 30 min at 37 °C in the absence of light.

Following the staining period, cells were washed with PBS to eliminate residual fluorescent dye before flow cytometric acquisition on a BD FACSCanto™ II instrument (BD Biosciences, San Jose, CA, USA) using excitation and emission settings of 488 and 525 nm, respectively. ROS measurements were normalized to untreated spheroid controls and reported as fold-change values.

#### 2.10.7. Assessment of Spheroid Viability After NAC-Mediated ROS Suppression

The influence of ROS suppression on treatment responsiveness was assessed using the CellTiter-Glo^®^ 3D Cell Viability Assay (Promega). At the completion of the treatment period, CellTiter-Glo^®^ reagent was added to each well at a 1:1 volume ratio to facilitate spheroid lysis and ATP extraction. Luminescent signals were then recorded with a microplate reader (BioTek Instruments). Viability data were calculated relative to untreated spheroid controls and presented as percentages.

The extent to which NAC pretreatment attenuated intracellular ROS accumulation and partially restored spheroid viability was interpreted as a functional indicator supporting the involvement of oxidative stress in mediating the cytotoxic effects associated with GA + CARB treatment in 3D tumor spheroids.

### 2.11. Bioinformatic Network and Pathway Enrichment Analyses

An integrative bioinformatic workflow was employed to investigate molecular networks potentially associated with the anticancer effects of GA and CARB in cervical cancer. Potential target genes associated with GA and CARB were compiled through searches of the SwissTargetPrediction database (http://www.swisstargetprediction.ch; accessed on 3 May 2026) and the PharmMapper platform (http://www.lilab-ecust.cn/pharmmapper; accessed on 3 May 2026). To generate a cervical cancer-related gene dataset, disease-associated genes were collected from GeneCards (https://www.genecards.org; accessed on 3 May 2026), DisGeNET (https://www.disgenet.org; accessed on 3 May 2026), and Online Mendelian Inheritance in Man (OMIM; https://www.omim.org; accessed on 3 May 2026).

Common targets shared between drug-associated proteins and cervical cancer-related genes were identified through intersection analysis using Venn diagram-based comparison. The resulting overlapping gene set was subsequently used for protein–protein interaction (PPI) network construction. PPI networks were constructed using the STRING database (https://string-db.org; accessed on 3 May 2026), applying an interaction confidence threshold of ≥0.7 to minimize low-confidence associations and improve network reliability. The resulting networks were subsequently imported into Cytoscape version 3.10 (Cytoscape Consortium, San Diego, CA, USA) for visualization and topological analysis.

To further explore the potential biological significance of the identified target networks, gene ontology (GO) functional enrichment and Kyoto Encyclopedia of Genes and Genomes (KEGG) pathway analyses were performed using the clusterProfiler package (version 4.8) within the R statistical environment. Enrichment analyses were interpreted to identify biological processes and signaling pathways potentially associated with oxidative stress regulation, mitochondrial dysfunction, apoptosis, and cell cycle-related mechanisms. Statistical significance thresholds for enrichment analyses were defined as *p* < 0.05 and false discovery rate-adjusted q < 0.05.

### 2.12. Statistical Analysis

All experimental procedures were performed using at least three independent biological replicates unless otherwise specified. The results are expressed as the mean ± standard deviation (SD), and all statistical calculations were carried out using GraphPad Prism version 9.0 (GraphPad Software Inc., San Diego, CA, USA).

Comparisons among multiple experimental groups were performed using one-way analysis of variance (ANOVA) followed by Tukey’s post hoc multiple comparison test for pairwise group evaluations. For experiments involving direct comparison between two groups, an unpaired Student’s *t*-test was applied where appropriate. Statistical significance was defined as *p* < 0.05.

For flow cytometry-based apoptosis, ROS, ΔΨm, and cell cycle analyses, representative experiments from at least three independent replicates were quantified and analyzed statistically. RT-qPCR experiments were performed using three independent biological replicates together with technical triplicates to minimize experimental variability.

CI analyses were calculated according to the Chou–Talalay method using CompuSyn software (Version 1.0, ComboSyn Inc., Paramus, NJ, USA). CI values were interpreted as follows: CI < 1 indicated synergistic interaction, CI = 1 indicated additive interaction, and CI > 1 indicated antagonistic interaction. Because CI analysis represents a mathematical modeling approach rather than inferential statistical testing, CI values were interpreted descriptively and were not subjected to additional statistical hypothesis testing.

For three-dimensional (3D) spheroid experiments, quantitative analyses of spheroid diameter, ATP-based viability, intracellular ROS accumulation, and live/dead fluorescence intensity distributions were performed using data obtained from at least three independent biological replicates. Image acquisition parameters were maintained identically across all experimental groups to reduce technical variability during fluorescence-based analyses.

## 3. Results

### 3.1. Cytotoxic Effects of GA and CARB on HeLa and HaCaT Cells

**Figure 1:**
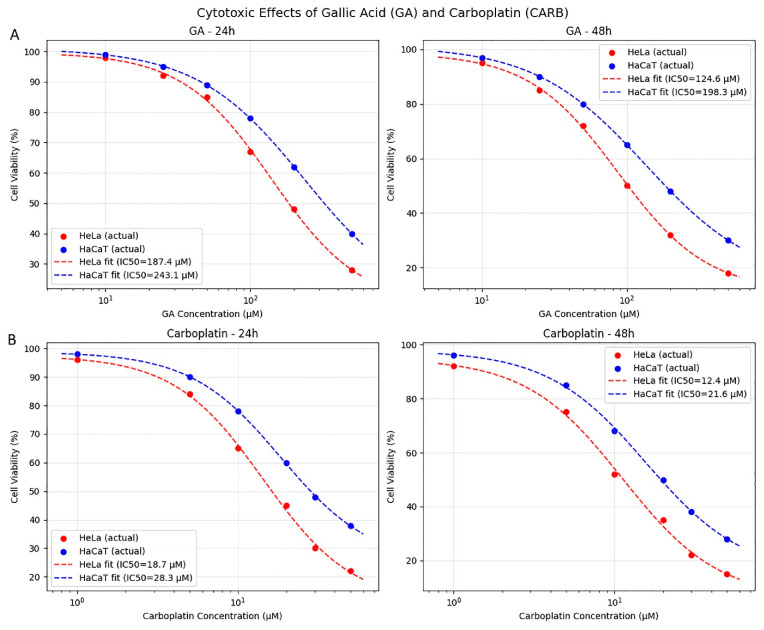
The effects of increasing GA and CARB concentrations on HeLa and HaCaT cell viability at 24 h and 48 h. Cellular metabolic activity was evaluated by the MTT assay following treatment with the indicated concentrations of each compound. (**A**) GA treatment reduced cell viability in both cell lines with lower IC_50_ values observed in HeLa cells compared with HaCaT cells. (**B**) CARB exposure similarly induced concentration- and time-dependent cytotoxicity, with HeLa cells demonstrating greater sensitivity relative to HaCaT cells. Data are presented as the mean ± SD (*n* = 3). Dose–response curves were generated by nonlinear regression analysis using a sigmoidal dose–response model.

The cytotoxic effects of GA and CARB were initially evaluated in HeLa cervical cancer cells and HaCaT immortalized keratinocytes using the MTT assay following 24 h and 48 h of exposure. Both agents reduced cell viability in a concentration- and time-dependent manner in both cell lines (Figure 1). Overall, prolonged exposure for 48 h resulted in a more pronounced decline in viability compared with 24 h treatment conditions.

GA treatment progressively decreased cellular viability across the tested concentration range in both HeLa and HaCaT cells (Figure 1A). Nonlinear regression analysis demonstrated IC_50_ values of 187.4 ± 12.3 µM and 124.6 ± 9.8 µM in HeLa cells at 24 h and 48 h, respectively. In HaCaT cells, the corresponding IC_50_ values were calculated as 243.1 ± 15.7 µM and 198.3 ± 11.2 µM. The lower IC_50_ values observed in HeLa cells were consistent with greater susceptibility of malignant cells to GA treatment relative to nontumoral keratinocytes.

Similarly, CARB exposure induced a marked reduction in cell viability in both cell lines in a dose- and time-dependent manner (Figure 1B). IC_50_ values in HeLa cells were determined as 18.7 ± 2.1 µM at 24 h and 12.4 ± 1.6 µM at 48 h, whereas HaCaT cells exhibited IC_50_ values of 28.3 ± 3.4 µM and 21.6 ± 2.8 µM at the respective time points. Comparable to GA treatment, HeLa cells displayed increased sensitivity to CARB relative to HaCaT cells, particularly following prolonged exposure periods.

Collectively, these findings demonstrated that both GA and CARB exert substantial cytotoxic activity in HeLa cells while showing comparatively lower toxicity in HaCaT cells, supporting the rationale for subsequent combination treatment experiments.

### 3.2. Evaluation of GA + CARB Combination Interaction

**Figure 2:**
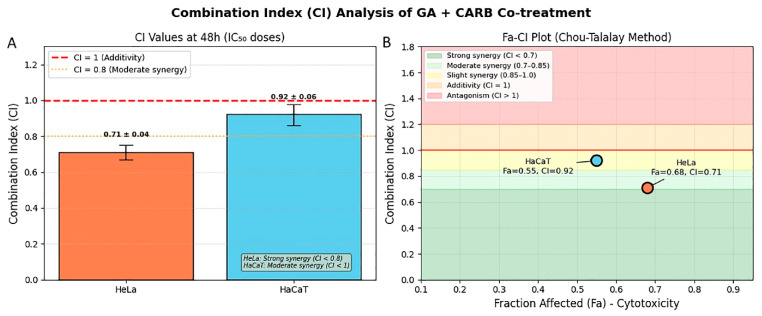
CI analysis of GA and CARB co-treatment in HeLa and HaCaT cells following 48 h exposure. (**A**) Quantitative CI values calculated according to the Chou–Talalay method. The red dashed line represents CI = 1 (additive interaction), whereas lower CI values indicate increasing synergistic interaction. HeLa cells exhibited a lower CI value (0.71 ± 0.04) compared with HaCaT cells (0.92 ± 0.06), consistent with a stronger combinational effect in malignant cells. (**B**) Fraction affected (Fa) versus CI distribution plot illustrating interaction profiles across predefined synergy regions. HeLa cells demonstrated greater treatment responsiveness together with lower CI values relative to HaCaT cells. Data are presented as mean ± SD (*n* = 3). CI, combination index; Fa, fraction affected.

To investigate whether combined exposure to GA and CARB enhances cytotoxic activity, HeLa and HaCaT cells were treated with IC_25_- and IC_50_-based combination regimens for 48 h, and drug interaction profiles were evaluated using the Chou–Talalay method (Figure 2).

CI analysis demonstrated differential interaction profiles between malignant and nontumoral cells. In HeLa cells, the GA + CARB combination yielded a CI value of 0.71 ± 0.04 at 48 h, consistent with a moderate synergistic interaction (Figure 2A). In contrast, HaCaT cells exhibited a CI value of 0.92 ± 0.06, indicating a substantially weaker combinational effect approaching additivity.

Fraction affected (Fa)-based CI distribution analysis further supported these findings (Figure 2B). HeLa cells demonstrated greater treatment responsiveness (Fa = 0.68) together with lower CI values relative to HaCaT cells (Fa = 0.55), suggesting enhanced susceptibility of cervical cancer cells to the combination regimen.

Cumulatively, these findings indicate that the GA + CARB combination exerts a more favorable combinational cytotoxic profile in HeLa cells compared with nontumoral keratinocytes, supporting subsequent mechanistic analyses of combination-mediated anticancer activity.

### 3.3. Induction of Apoptotic Cell Death by GA and CARB Combination Treatment

**Figure 3:**
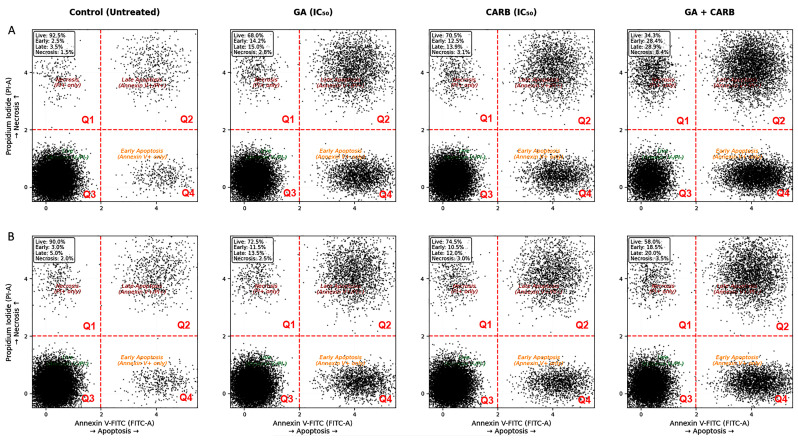
Flow cytometric Annexin V-FITC/PI dot plot analysis of HeLa (**A**) and HaCaT (**B**) cells after 48 h treatment with GA (IC_50_), CARB (IC_50_), or the GA + CARB combination. Cell distributions are shown within four quadrants: Q3, viable cells (Annexin V^−^/PI^−^); Q4, early apoptotic cells (Annexin V^+^/PI^−^); Q2, late apoptotic cells (Annexin V^+^/PI^+^); and Q1, necrotic cells (Annexin V^−^/PI^+^). Among HeLa cells, the combined treatment produced the greatest shift toward apoptosis, as evidenced by increased early and late apoptotic fractions accompanied by a marked decline in the viable cell population compared with the control and single-treatment groups. A similar but less pronounced apoptotic pattern was observed in HaCaT cells. Quantitative quadrant distributions demonstrated that total apoptotic cell populations (early + late apoptosis) increased from 4.5% in untreated HeLa cells to 57.3% following GA + CARB treatment, whereas total apoptosis in HaCaT cells increased from 5.0% to 38.5% under identical treatment conditions. Necrotic cell populations remained comparatively limited relative to apoptotic fractions, indicating that apoptosis represented the predominant mode of treatment-associated cell death. Percentages shown within each quadrant represent representative distribution profiles obtained from three independent experiments. Red dashed lines indicate quadrant boundaries separating viable, apoptotic, and necrotic cell populations.

Annexin V-FITC/PI double staining followed by flow cytometric analysis was performed to evaluate treatment-associated apoptotic cell death after 48 h of exposure (Figure 3). Both GA and CARB monotherapies increased apoptotic cell populations relative to untreated controls in HeLa and HaCaT cells.

In HeLa cells, untreated controls predominantly consisted of viable cells, whereas GA and CARB exposure resulted in clear increases in both early and late apoptotic populations (Figure 3A). Total apoptotic cell percentages increased from 4.5% in control cells to 29.2% following GA treatment and 26.4% following CARB treatment. Notably, the GA + CARB combination induced the most pronounced apoptotic response, increasing total apoptosis to 57.3%, which was substantially greater than that observed with either monotherapy.

A similar but less pronounced pattern was observed in HaCaT cells (Figure 3B). Total apoptotic cell populations increased from 5.0% in untreated controls to 24.8% and 22.3% following GA and CARB exposure, respectively. Combination treatment increased total apoptosis to 35.5%, remaining lower than the apoptotic response observed in HeLa cells under identical treatment conditions.

Although modest increases in necrotic cell populations were detected following combination treatment, apoptotic cell death represented the predominant mode of cytotoxicity in both cell lines. In combination, these findings suggest that the GA + CARB combination enhances apoptosis more effectively in HeLa cervical cancer cells than in nontumoral keratinocytes.

### 3.4. Functional Evaluation of ROS-Associated Cytotoxicity Using NAC Rescue

**Figure 4:**
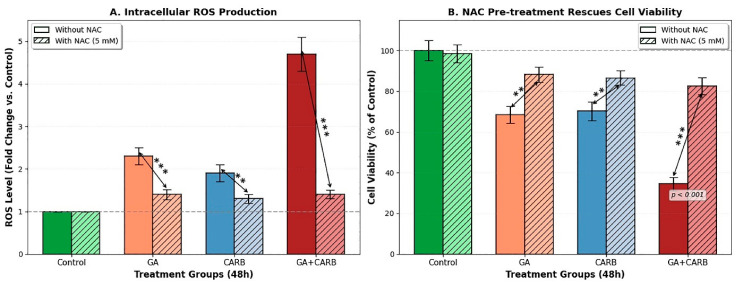
The influence of NAC on intracellular ROS generation and cellular viability in HeLa cells treated with GA, CARB, or the GA + CARB combination for 48 h. Prior to drug exposure, cells received a 1 h incubation with 5 mM NAC. (**A**) Intracellular ROS levels assessed by the DCFH-DA assay and expressed as fold-change values relative to untreated controls. The greatest increase in ROS was observed following combined treatment, whereas NAC administration markedly reduced this response. (**B**) Viability analysis performed using the MTT assay. Exposure to the GA + CARB combination resulted in a pronounced reduction in viable cells, while antioxidant intervention partially reversed this effect. Solid bars denote treatment groups without NAC, and hatched bars represent NAC-treated groups. Values are presented as the mean ± SD (*n* = 3). ** *p* < 0.01 and *** *p* < 0.001 versus the corresponding untreated control groups.

Intracellular ROS production following GA, CARB, and GA + CARB treatment was evaluated using the DCFH-DA fluorescence assay after 48 h of exposure (Figure 4A). Both GA and CARB monotherapies moderately increased intracellular ROS accumulation relative to untreated controls, producing approximately 2.3-fold and 1.9-fold elevations, respectively. Notably, combination treatment induced the highest ROS increase, reaching approximately 4.7-fold above control levels, which was markedly greater than the ROS elevations observed in single-agent groups.

To further investigate the functional contribution of oxidative stress to treatment-associated cytotoxicity, antioxidant rescue experiments were performed using NAC. Pretreatment with 5 mM NAC for 1 h substantially attenuated intracellular ROS accumulation induced by the GA + CARB combination, reducing ROS levels to approximately 1.4-fold relative to untreated controls (Figure 4A).

In parallel with ROS suppression, NAC pretreatment partially restored cellular viability in GA + CARB-treated cells (Figure 4B). While combination treatment alone reduced viability to 34.5 ± 3.2% of control values, NAC exposure increased viability to 82.5 ± 4.1%, indicating a marked reduction in treatment-associated cytotoxicity following ROS scavenging.

In combination, these findings support the involvement of ROS-associated oxidative stress in mediating the cytotoxic effects induced by the GA + CARB combination in HeLa cells.

### 3.5. Mitochondrial Membrane Depolarization Following GA and CARB Treatment

**Figure 5:**
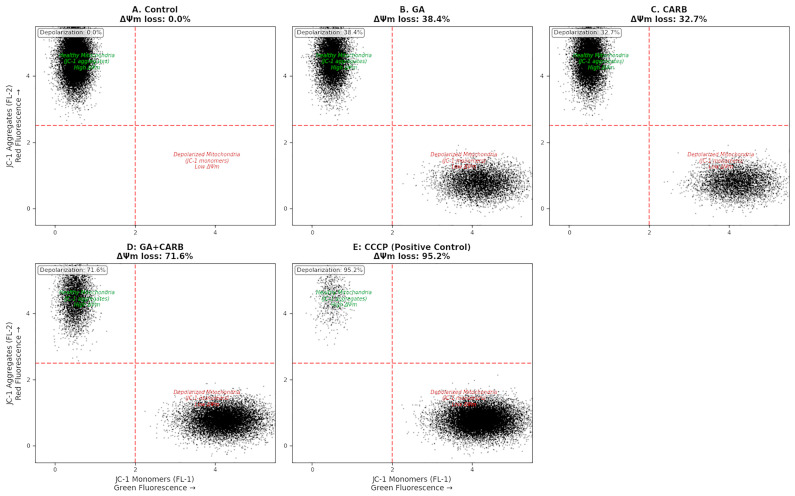
Assessment of ΔΨm in HeLa cells by JC-1 fluorescence staining after 48 h of treatment with GA (IC_50_), CARB (IC_50_), or the GA + CARB combination. CCCP (50 µM, 30 min) served as a positive control for mitochondrial membrane depolarization. Representative flow cytometry plots display the relative distribution of JC-1 aggregates (red fluorescence, polarized mitochondria) and JC-1 monomers (green fluorescence, depolarized mitochondria). Red dashed lines indicate quadrant boundaries. (**A**) Untreated control cells demonstrating preserved mitochondrial polarization. (**B**) GA-treated cells showing 38.4% ΔΨm loss. (**C**) CARB-treated cells showing 32.7% ΔΨm loss. (**D**) GA + CARB-treated cells demonstrating marked mitochondrial depolarization with 71.6% ΔΨm loss. (**E**) CCCP-treated positive control cells exhibiting extensive mitochondrial membrane depolarization (95.2% ΔΨm loss). ΔΨm, mitochondrial membrane potential; CCCP, carbonyl cyanide m-chlorophenyl hydrazone.

Changes in ΔΨm were evaluated using JC-1 fluorescence staining followed by flow cytometric analysis in HeLa cells after 48 h of treatment (Figure 5). In healthy mitochondria, JC-1 accumulates as red fluorescent aggregates, whereas mitochondrial depolarization promotes formation of green fluorescent monomers. Therefore, increased green fluorescence together with reduced red fluorescence was interpreted as an indicator of impaired mitochondrial membrane integrity.

Both GA and CARB monotherapies induced measurable mitochondrial depolarization relative to untreated control cells. GA treatment produced approximately 38.4 ± 3.1% ΔΨm loss, whereas CARB exposure resulted in 32.7 ± 2.8% mitochondrial depolarization (Figure 5B,C). Notably, the GA + CARB combination induced substantially greater mitochondrial membrane disruption, increasing ΔΨm loss to 71.6 ± 4.3%, which was markedly higher than that observed in either monotherapy group (Figure 5D).

As expected, treatment with the mitochondrial uncoupling agent CCCP (50 µM, 30 min) induced extensive mitochondrial depolarization and served as a positive control for ΔΨm disruption (Figure 5E).

Viewed together, these findings indicate that combined GA + CARB exposure markedly impairs mitochondrial membrane integrity, consistent with enhanced mitochondrial dysfunction during treatment-associated cell death.

### 3.6. Effects of GA and CARB Combination on Cell Cycle Distribution

**Figure 6:**
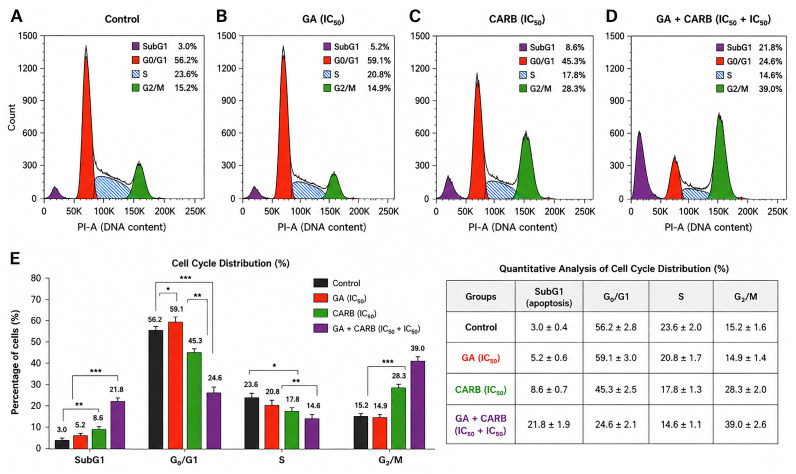
The effects of GA, CARB, and the GA + CARB combination on cell cycle distribution in HeLa cells following 48 h treatment. Cells were stained with PI and analyzed by flow cytometry. (**A**–**D**) Representative DNA content histograms demonstrating the distribution of cell populations within SubG1, G_0_/G_1_, S, and G_2_/M phases following treatment with GA (IC_50_), CARB (IC_50_), or the GA + CARB combination. (**A**) Untreated control cells displayed predominant accumulation within the G_0_/G_1_ phase with minimal SubG1 distribution. (**B**) GA treatment induced a modest increase in G_0_/G_1_-phase accumulation. (**C**) CARB treatment promoted enrichment of the G_2_/M population. (**D**) Combination treatment markedly increased both SubG1 and G_2_/M fractions while reducing G_0_/G_1_- and S-phase populations. (**E**) Quantitative analysis of cell cycle phase distributions. GA + CARB treatment significantly increased SubG1-associated apoptotic DNA fragmentation and G_2_/M accumulation relative to control and monotherapy groups. Data are presented as the mean ± SD (*n* = 3). Statistical analysis was performed using one-way ANOVA followed by Tukey’s post hoc test. * *p* < 0.05, ** *p* < 0.01, and *** *p* < 0.001.

To further investigate whether the cytotoxic effects associated with GA + CARB treatment were accompanied by alterations in cell cycle progression, PI-based flow cytometric cell cycle analysis was performed following 48 h of exposure in HeLa cells (Figure 6).

Untreated control cells predominantly accumulated within the G_0_/G_1_ phase (56.2 ± 2.8%), followed by S phase (23.6 ± 2.0%) and G_2_/M phase (15.2 ± 1.6%), whereas the SubG1 fraction remained minimal (3.0 ± 0.4%), indicating low basal apoptotic DNA fragmentation.

GA treatment induced a modest increase in the G_0_/G_1_ population to 59.1 ± 3.0% together with a reduction in S-phase cells to 20.8 ± 1.7% (Figure 6B,E). In contrast, CARB exposure promoted the accumulation of cells within the G_2_/M phase, increasing the G_2_/M population to 28.3 ± 2.0%, accompanied by a reduction in G_0_/G_1_-phase cells relative to untreated controls (Figure 6C,E). CARB-treated cells additionally demonstrated a moderate increase in the SubG1 fraction (8.6 ± 0.7%).

Notably, the GA + CARB combination produced the most pronounced redistribution of cell cycle populations (Figure 6D,E). Combination treatment markedly increased the SubG1 population to 21.8 ± 1.9% together with substantial G_2_/M accumulation (39.0 ± 2.6%). In parallel, G_0_/G_1_-phase cells decreased to 24.6 ± 2.1%, whereas the S-phase fraction was reduced to 14.6 ± 1.1%.

In summary, these findings indicate that combined GA + CARB exposure profoundly disrupts normal cell cycle progression in HeLa cells, promoting G_2_/M-phase accumulation together with increased SubG1-associated apoptotic DNA fragmentation.

### 3.7. Effects of GA and CARB Combination on Apoptosis- and Cell Cycle-Related Gene Expression

**Figure 7:**
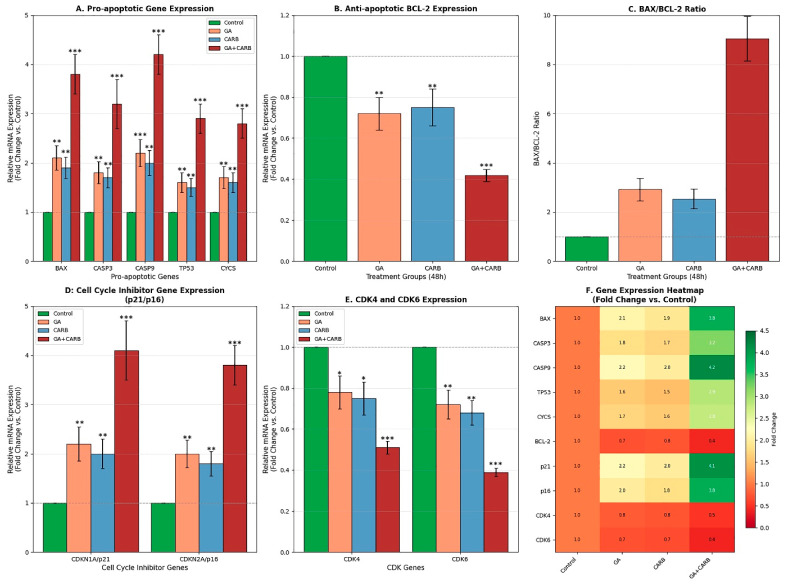
The effects of GA, CARB, and the GA + CARB combination on apoptosis- and cell cycle-related gene expression in HeLa cells following 48 h treatment. Relative mRNA expression levels were determined by RT-qPCR and normalized to housekeeping genes. (**A**) The expression profiles of pro-apoptotic genes including *BAX, CASP3, CASP9, TP53*, and *CYCS*. Combination treatment produced the highest expression levels among all experimental groups. (**B**) The relative expression of the anti-apoptotic gene *BCL-2*. (**C**) Quantitative analysis of the *BAX/BCL-2* ratio following treatment. (**D**) The expression levels of cell cycle inhibitory genes *CDKN1A/p21* and *CDKN2A/p16*. (**E**) Relative expression of *CDK4* and *CDK6* following treatment exposure. (**F**) A heatmap summarizing global gene expression alterations across all treatment groups. Green coloration represents relative upregulation, whereas red coloration indicates relative downregulation compared with untreated controls. Data are presented as the mean ± SD (*n* = 3). Statistical analysis was performed using one-way ANOVA followed by Tukey’s post hoc test. * *p* < 0.05, ** *p* < 0.01, and *** *p* < 0.001 versus the control group.

To further investigate molecular alterations associated with GA + CARB-mediated cytotoxicity, the expression profiles of apoptosis- and cell cycle-related genes were evaluated in HeLa cells by RT-qPCR following 48 h of treatment (Figure 7).

Combination treatment markedly increased the expression of multiple pro-apoptotic genes relative to untreated controls (Figure 7A). Specifically, GA + CARB exposure elevated *BAX*, *CASP3, CASP9, TP53*, and *CYCS mRNA* expression levels to 3.8 ± 0.4-fold, 3.2 ± 0.5-fold, 4.2 ± 0.4-fold, 2.9 ± 0.3-fold, and 2.8 ± 0.3-fold of control values, respectively. In parallel, expression of the anti-apoptotic gene *BCL-2* was reduced to 0.42 ± 0.03-fold relative to untreated cells (Figure 7B). Consistent with these findings, the *BAX/BCL-2* ratio was substantially increased in the combination group compared with control and monotherapy groups (Figure 7C).

Expression analysis of cell cycle-associated regulatory genes demonstrated that GA + CARB treatment significantly increased *CDKN1A/p21* and *CDKN2A/p16* expression levels to 4.1 ± 0.6-fold and 3.8 ± 0.4-fold, respectively (Figure 7D). In contrast, expression of *CDK4* and *CDK6* was reduced to 0.51 ± 0.03-fold and 0.39 ± 0.02-fold of control values following combination exposure (Figure 7E).

Although GA and CARB monotherapies also induced detectable transcriptional alterations, the magnitude of these changes remained lower than that observed in the combination group. A global overview of treatment-associated gene expression profiles is presented in the heatmap analysis (Figure 7F), in which green coloration indicates relative upregulation and red coloration indicates relative downregulation compared with untreated controls.

Taken together, these findings demonstrate that the GA + CARB combination is associated with coordinated transcriptional changes involving pro-apoptotic signaling and cell cycle regulatory pathways in HeLa cells.

### 3.8. Active Caspase-3 Expression Following GA and CARB Treatment 

**Figure 8:**
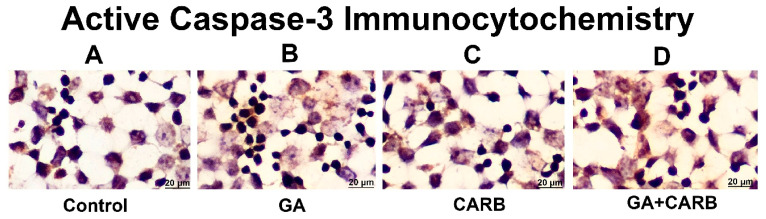
Immunocytochemical evaluation of active caspase-3 expression in HeLa cells following 48 h of exposure to GA (IC_50_), CARB (IC_50_), or the GA + CARB combination. Active caspase-3 immunoreactivity was visualized using DAB chromogen staining and appeared as brown cytoplasmic staining, whereas nuclei were counterstained blue-purple with hematoxylin. (**A**) Untreated control cells exhibited minimal basal active caspase-3 immunoreactivity. (**B**) GA-treated cells demonstrated increased cytoplasmic DAB positivity relative to controls. (**C**) CARB-treated cells similarly showed enhanced active caspase-3 staining. (**D**) GA + CARB-treated cells displayed the strongest immunoreactivity, characterized by diffuse and intense cytoplasmic brown staining together with increased numbers of DAB-positive cells. Semi-quantitative H-score analysis demonstrated markedly elevated active caspase-3 expression in the combination group relative to control and monotherapy groups. Representative images were obtained from three independent experiments. Scale bar: 20 μm.

Immunocytochemical staining was performed to assess treatment-associated alterations in active caspase-3 protein expression in HeLa cells following 48 h of exposure to GA, CARB, or the GA + CARB combination (Figure 8). Active caspase-3 immunoreactivity was visualized as brown cytoplasmic DAB staining, whereas nuclei were counterstained blue-purple with hematoxylin.

Untreated control cells exhibited minimal basal DAB positivity together with weak active caspase-3 immunoreactivity (Figure 8A). In contrast, both GA and CARB monotherapies induced visibly increased cytoplasmic staining intensity relative to control cells, indicating elevated active caspase-3 expression following treatment exposure (Figure 8B,C).

**Figure 9:**
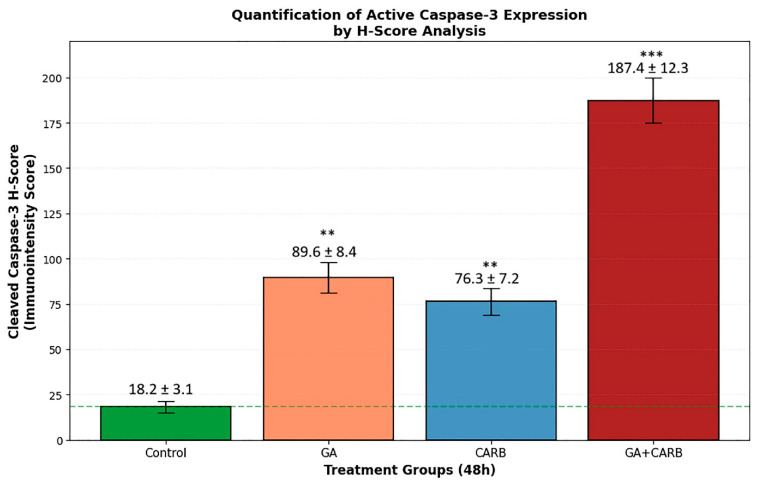
Semi-quantitative H-score analysis of active caspase-3 immunoreactivity in HeLa cells following 48 h of exposure to GA, CARB, or the GA + CARB combination. Cells were immunostained for cleaved caspase-3 using DAB chromogen staining, and staining intensity was quantified using the H-score method. The GA + CARB combination produced the highest active caspase-3 H-score (187.4 ± 12.3), compared with control cells (18.2 ± 3.1), GA-treated cells (89.6 ± 8.4), and CARB-treated cells (76.3 ± 7.2). Combination treatment demonstrated significantly greater active caspase-3 immunoreactivity relative to both control and monotherapy groups. No statistically significant difference was observed between GA and CARB monotherapies. Data are presented as mean ± SD (*n* = 3). ** *p* < 0.01 and *** *p* < 0.001 versus control group; ns, not significant.

The most prominent immunoreactivity was observed in the GA + CARB combination group (Figure 8D). Combination-treated cells demonstrated diffuse and intense cytoplasmic brown staining together with an increased proportion of DAB-positive cells compared with both untreated and monotherapy groups. Semi-quantitative H-score analysis further supported these observations (Figure 9). The active caspase-3 H-score increased from 18.2 ± 3.1 in untreated controls to 89.6 ± 8.4 following GA treatment and 76.3 ± 7.2 following CARB treatment, whereas the GA + CARB combination produced the highest H-score value (187.4 ± 12.3).

Considered as a whole, these findings indicate that combined GA + CARB exposure is associated with markedly enhanced active caspase-3 immunoreactivity relative to single-agent treatments, consistent with increased apoptotic activity in HeLa cells.

### 3.9. Effects of GA + CARB Treatment on 3D Tumor Spheroid Growth and Viability

**Figure 10:**
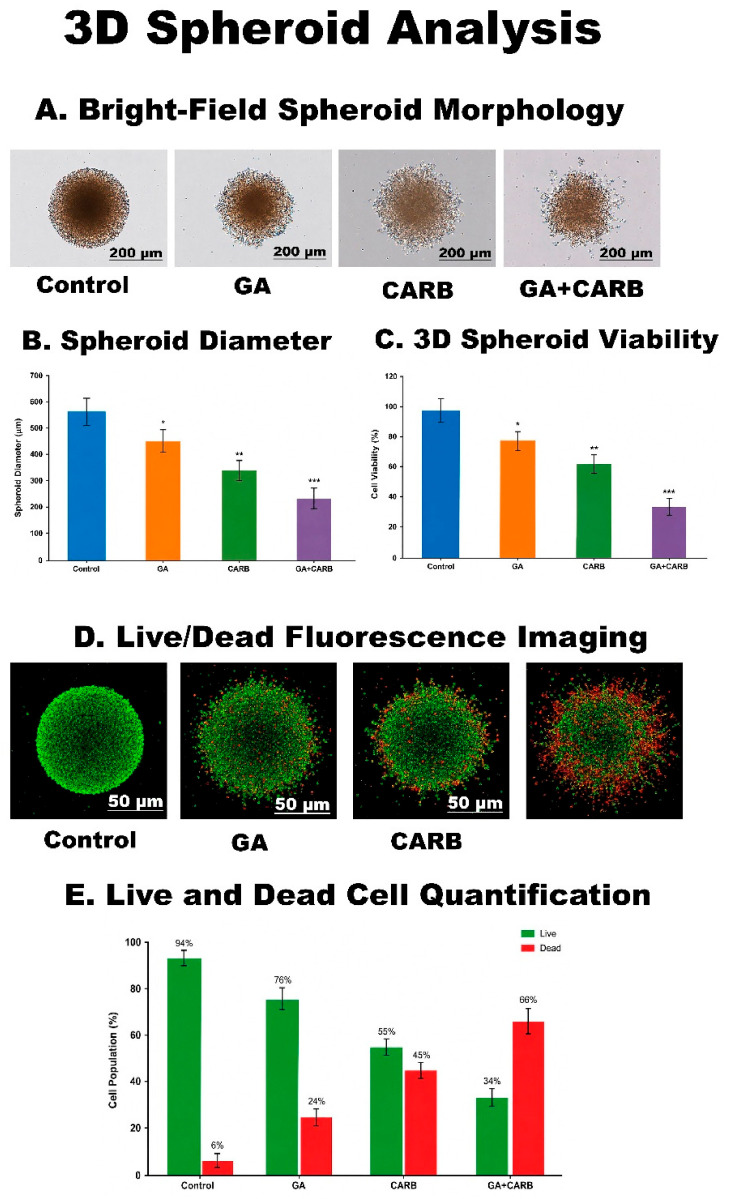
Three-dimensional (3D) spheroid analysis of HeLa cells following treatment with GA, CARB, or the GA + CARB combination for 72 h. (**A**) Representative bright-field images of spheroid morphology. Untreated control spheroids retained compact spherical architecture with smooth borders and dense cellular organization. GA treatment induced mild structural loosening, whereas CARB exposure caused more pronounced border irregularities and reduced spheroid integrity. Combination treatment produced marked spheroid disorganization characterized by irregular morphology, reduced compactness, and peripheral cellular dispersion. Scale bar: 200 μm. (**B**) Quantitative analysis of spheroid diameter. Both monotherapies reduced spheroid size relative to untreated controls, whereas the GA + CARB combination produced the greatest reduction in spheroid diameter. (**C**) ATP-based viability analysis of 3D spheroids. Combination treatment induced the strongest reduction in spheroid viability compared with control and monotherapy groups. (**D**) Representative live/dead fluorescence images of spheroids stained with Calcein-AM (green, viable cells) and Ethidium homodimer-1 (red, non-viable cells). Control spheroids predominantly exhibited green fluorescence, whereas combination-treated spheroids demonstrated extensive red fluorescence together with marked disruption of spheroid architecture. Scale bar: 50 μm. (**E**) Quantitative analysis of live and dead cell populations within spheroids. Combination treatment resulted in the highest proportion of non-viable cells together with the lowest viable cell fraction among all experimental groups. Data are presented as the mean ± SD from three independent experiments (*n* = 3). Statistical analysis was performed using one-way ANOVA followed by Tukey’s post hoc test. * *p* < 0.05, ** *p* < 0.01, and *** *p* < 0.001 versus untreated control.

To further evaluate treatment-associated responses under physiologically relevant tumor-like conditions, a three-dimensional (3D) HeLa spheroid model was established (Figure 10). Untreated control spheroids retained a compact spherical morphology characterized by smooth borders and dense cellular organization throughout the experimental period (Figure 10A). In contrast, GA-treated spheroids exhibited mild structural loosening and partial loss of compactness, whereas CARB exposure induced more evident border irregularities together with reduced spheroid integrity. These morphological alterations were most pronounced in the GA + CARB combination group, in which spheroids demonstrated marked architectural disruption, irregular morphology, and peripheral cellular dispersion consistent with impaired spheroid cohesion.

Quantitative analysis demonstrated that both GA and CARB monotherapies significantly reduced spheroid diameter relative to untreated controls (Figure 10B). Notably, combination treatment produced the greatest reduction in spheroid size compared with both monotherapy groups, indicating enhanced suppression of spheroid growth under diffusion-limited 3D culture conditions.

ATP-based viability analysis further demonstrated that GA and CARB monotherapies moderately decreased spheroid viability, whereas the GA + CARB combination induced the strongest reduction in viable cell populations, decreasing viability to approximately 30–40% of untreated control values (Figure 10C).

Consistent with these findings, live/dead fluorescence imaging revealed predominantly viable cell populations in untreated spheroids, as indicated by intense green fluorescence signals (Figure 10D). GA-treated spheroids displayed limited accumulation of non-viable cells, whereas CARB treatment produced more extensive red fluorescence distribution. The GA + CARB combination generated the highest proportion of red fluorescent non-viable cells together with substantial disruption of spheroid architecture.

Quantitative live/dead analysis further confirmed these observations (Figure 10E). While untreated spheroids consisted predominantly of viable cells, monotherapy groups demonstrated moderate increases in dead cell populations. In contrast, the GA + CARB combination produced the lowest viable cell fraction together with the highest proportion of non-viable cells among all treatment groups.

Across all experimental analyses, these findings demonstrate that combined GA + CARB exposure exerts substantially greater anti-spheroid activity than either monotherapy alone, resulting in impaired spheroid integrity, reduced viability, and increased treatment-associated cell death in 3D HeLa tumor spheroids.

### 3.10. Functional Evaluation of ROS Contribution in 3D Tumor Spheroids Using NAC Rescue

**Figure 11:**
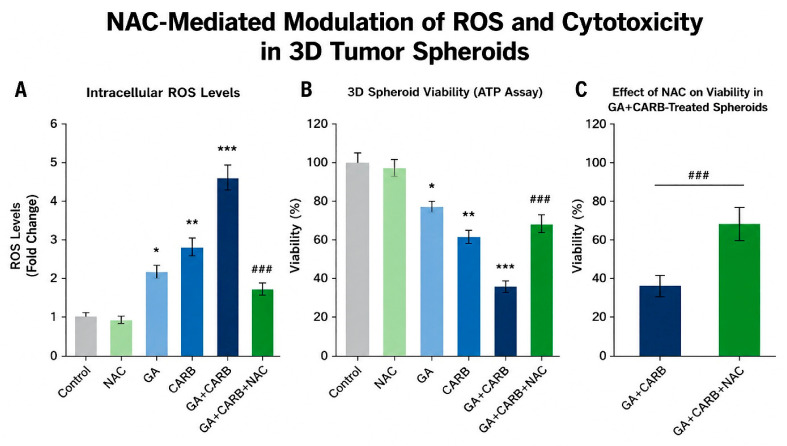
NAC-mediated modulation of ROS accumulation and spheroid viability in 3D HeLa tumor spheroids. (**A**) Intracellular ROS levels were quantified using DCFH-DA fluorescence following treatment with GA, CARB, GA + CARB, or GA + CARB in the presence of NAC pretreatment. GA and CARB monotherapies moderately increased ROS production compared with untreated control spheroids, whereas the GA + CARB combination induced the highest ROS accumulation (~4.5-fold above control). NAC pretreatment substantially attenuated ROS elevation in the combination group, reducing fluorescence intensity toward near-basal levels. (**B**) ATP-based luminescence assay showing viability changes in 3D spheroids following treatment. GA and CARB alone moderately reduced spheroid viability, while the GA + CARB combination produced the strongest cytotoxic response (~35–40% viability relative to control). NAC pretreatment significantly restored spheroid viability in the combination-treated group, indicating partial reversal of treatment-associated cytotoxicity. (**C**) Direct comparison of viability between GA + CARB-treated spheroids and NAC-pretreated GA + CARB spheroids, demonstrating significant recovery of viable cell populations following ROS scavenging. Data are presented as the mean ± SD from three independent experiments (*n* = 3). * *p* < 0.05, ** *p* < 0.01, and *** *p* < 0.001 versus the untreated control; ### *p* < 0.001 versus the GA + CARB-treated group.

To investigate whether oxidative stress contributes to the cytotoxic effects observed in 3D spheroids, NAC-based antioxidant rescue experiments were performed. Intracellular ROS analysis demonstrated that GA and CARB monotherapies moderately increased ROS production compared with untreated spheroids (*p* < 0.05; Figure 11A). In contrast, the GA + CARB combination induced a marked elevation in ROS levels, reaching approximately 4–5-fold above control values (*p* < 0.001; Figure 11A). Pretreatment with NAC substantially attenuated ROS accumulation in the combination group, reducing fluorescence intensity to levels approaching those observed in untreated controls (Figure 11A).

This reduction in oxidative stress was accompanied by partial restoration of spheroid viability. ATP-based viability analysis demonstrated that NAC pretreatment significantly reversed the cytotoxic effects associated with GA + CARB exposure, resulting in a marked increase in viable spheroid cell populations compared with combination treatment alone (*p* < 0.001; Figure 11B). Direct comparison of the GA + CARB and GA + CARB + NAC groups further confirmed that ROS scavenging significantly restored spheroid viability following combination treatment (Figure 11C).

Morphologically, NAC-pretreated spheroids retained a more compact architecture and exhibited less structural disruption compared with spheroids treated with the GA + CARB combination alone. These findings support the involvement of ROS-associated oxidative stress in mediating the cytotoxic and spheroid-disruptive effects induced by GA + CARB treatment in 3D tumor spheroids.

### 3.11. Bioinformatics Analysis

#### 3.11.1. PPI Network Analysis

**Figure 12:**
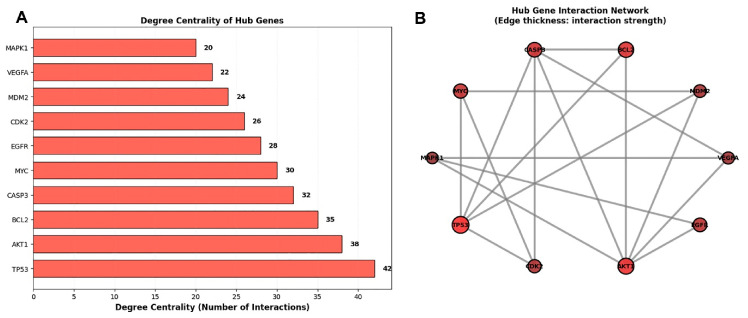
Bioinformatic hub gene interaction analysis associated with GA and CARB treatment. (**A**) Degree centrality ranking of the top hub genes identified by Cytoscape CytoHubba analysis following STRING-based PPI network construction. *TP53* exhibited the highest interaction connectivity, followed by *AKT1, BCL2, CASP3, MYC, EGFR, CDK2, MDM2, VEGFA,* and *MAPK1*. Degree centrality values represent the number of interaction-associated connections for each gene within the network. (**B**) A hub gene interaction subnetwork illustrating the interconnectivity among the top-ranked genes. Edge thickness reflects relative interaction strength between nodes. The interaction map suggests potential associations among apoptosis-, oxidative stress-, and cell cycle-related signaling components. As a bioinformatic prediction, these network relationships should be considered hypothesis-generating and require further biological validation.

To investigate the molecular interactions potentially associated with the anticancer activity of GA and CARB, PPI network analysis was performed using STRING-derived interaction data followed by Cytoscape-based network visualization and topological analysis (Figure 12).

Degree centrality analysis identified TP53, AKT1, BCL2, CASP3, MYC, EGFR, CDK2, MDM2, VEGFA, and MAPK1 as the top-ranked hub genes exhibiting the highest interaction connectivity within the network (Figure 12A). Among these genes, TP53 demonstrated the highest degree centrality score, followed by AKT1 and BCL2, indicating their central positions within the predicted interaction network.

Hub gene interaction mapping further demonstrated extensive associations among apoptosis-related, oxidative stress-associated, and cell cycle regulatory signaling components (Figure 12B). The observed interaction architecture suggests the potential involvement of multiple overlapping molecular pathways that may contribute to treatment-associated cellular responses.

From an integrated perspective, these findings suggest that GA- and CARB-associated targets participate in interconnected signaling networks relevant to apoptosis regulation, oxidative stress responses, and cervical cancer cell survival. However, these network-based associations are predictive in nature and should be considered hypothesis-generating observations that require further biological and functional validation.

#### 3.11.2. Functional Pathway Enrichment Analysis

**Figure 13:**
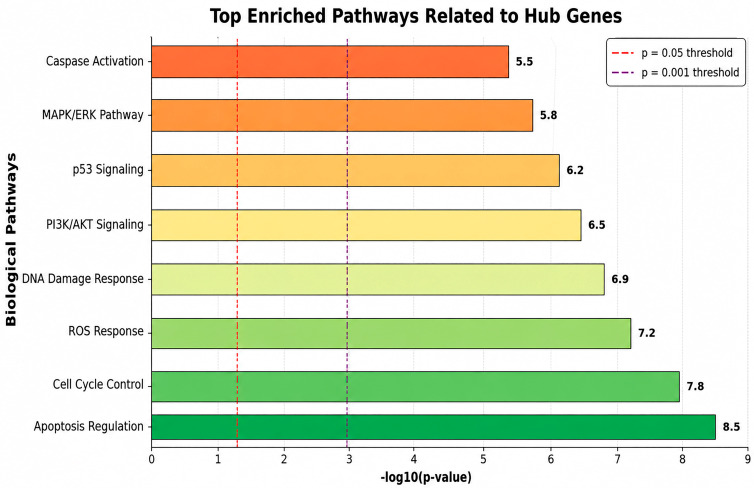
Functional enrichment analysis of hub genes showing significantly enriched biological pathways associated with GA and CARB treatment. Top enriched pathways included apoptosis regulation, cell cycle control, ROS response, DNA damage response, *PI3K/AKT* signaling, *p53* signaling, *MAPK/ERK* signaling, and caspase activation. Horizontal bars represent enrichment significance expressed as −log10 (*p*-value). Red dashed lines indicate the *p* = 0.05 significance threshold, whereas purple dashed lines indicate the *p* = 0.001 threshold. These enriched pathways suggest potential involvement of apoptosis regulation, oxidative stress signaling, and cell cycle-related molecular responses. As bioinformatic enrichment results, these pathway associations should be considered hypothesis-generating and require further biological and functional validation.

Functional pathway enrichment analysis demonstrated significant enrichment of multiple biological pathways associated with apoptosis regulation, oxidative stress responses, DNA damage signaling, and cell cycle control (Figure 13).

Among the most significantly enriched pathways were apoptosis regulation, cell cycle control, ROS response, DNA damage response, PI3K/AKT signaling, p53 signaling, MAPK/ERK signaling, and caspase activation pathways. These enrichment results suggest that the molecular targets associated with GA and CARB may be linked to multiple interconnected regulatory processes involved in cancer cell survival and stress adaptation. However, these pathway associations are based on bioinformatic predictions and should be considered hypothesis-generating observations requiring further biological and functional validation.

#### 3.11.3. GO Enrichment Analysis

**Figure 14:**
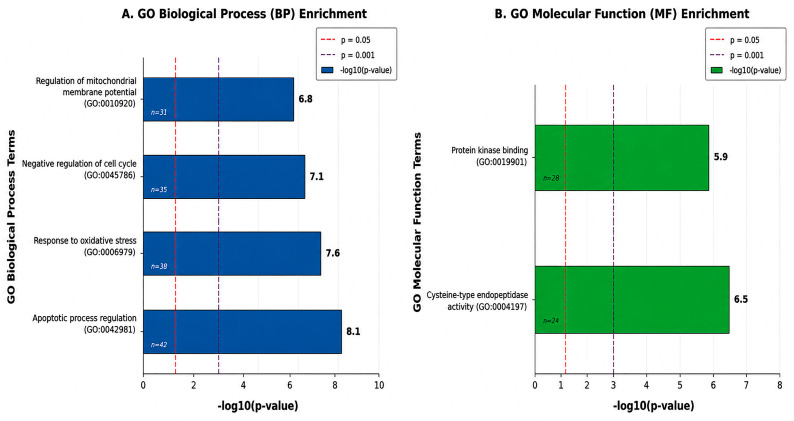
GO enrichment analysis of common target genes associated with GA and CARB treatment. (**A**) BP enrichment analysis demonstrating significant enrichment of apoptotic process regulation, response to oxidative stress, negative regulation of cell cycle progression, and regulation of ΔΨm. (**B**) MF enrichment analysis showing enrichment of cysteine-type endopeptidase activity and protein kinase binding. Horizontal bars represent enrichment significance expressed as −log10 (*p*-value). Red and purple dashed lines indicate *p* = 0.05 and *p* = 0.001 significance thresholds, respectively. These enriched GO terms suggest potential associations with apoptosis-, oxidative stress-, mitochondrial function-, and cell cycle-related biological processes. As bioinformatic enrichment results, these observations should be considered hypothesis-generating and require further biological and functional validation.

GO enrichment analysis further demonstrated significant enrichment in biological processes associated with apoptotic regulation, oxidative stress responses, mitochondrial dysfunction, and cell cycle inhibition (Figure 14).

Within the Biological Process (BP) category, regulation of apoptotic process (GO:0042981), response to oxidative stress (GO:0006979), negative regulation of cell cycle progression (GO:0045786), and regulation of ΔΨm (GO:0010920) were identified among the most enriched terms (Figure 14A).

In the Molecular Function (MF) category, cysteine-type endopeptidase activity involved in apoptotic signaling (GO:0004197) and protein kinase binding (GO:0019901) represented the most significantly enriched MFs (Figure 14B).

Collectively, these enrichment results suggest potential associations with apoptosis-associated proteolytic activity, oxidative stress-related signaling, and mitochondrial regulatory processes that may contribute to the observed treatment-associated cellular responses. However, as GO enrichment analyses are based on bioinformatic annotations and statistical overrepresentation, these findings should be considered hypothesis-generating and require further biological and functional validation.

#### 3.11.4. KEGG Pathway Enrichment Analysis

**Figure 15:**
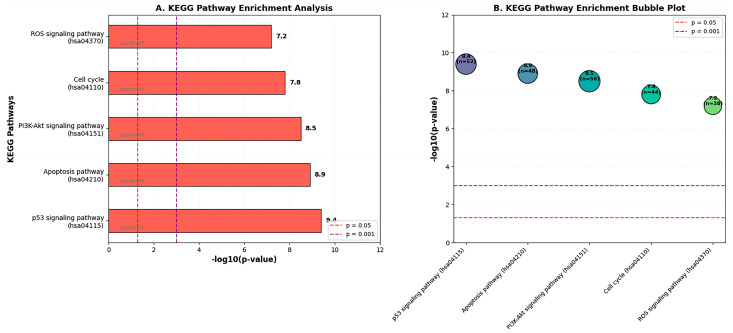
KEGG pathway enrichment analysis associated with GA and CARB treatment. (**A**) Bar plot showing significantly enriched pathways, including *p53* signaling, apoptosis, *PI3K-Akt* signaling, cell cycle regulation, and ROS-associated signaling pathways. Horizontal bars represent enrichment significance expressed as −log10 (*p*-value). (**B**) Bubble plot visualization of enriched KEGG pathways. Bubble size corresponds to the number of associated genes within each pathway, whereas vertical position represents enrichment significance (−log10 (*p*-value)). Red and purple dashed lines indicate *p* = 0.05 and *p* = 0.001 significance thresholds, respectively. These enriched pathways suggest potential associations with apoptosis regulation, oxidative stress-related signaling, DNA damage responses, and cell cycle control. As KEGG enrichment results are based on bioinformatic pathway annotations, these observations should be considered predictive and hypothesis-generating and require further biological and functional validation.

KEGG pathway enrichment analysis identified significant enrichment of signaling pathways associated with apoptosis, oxidative stress responses, DNA damage signaling, and cell cycle regulation (Figure 15).

The most significantly enriched pathways included the p53 signaling pathway (hsa04115), apoptosis pathway (hsa04210), PI3K-Akt signaling pathway (hsa04151), cell cycle pathway (hsa04110), and ROS-associated signaling pathway (hsa04370) (Figure 15A). Bubble plot visualization further demonstrated that these pathways contained substantial numbers of associated target genes and exhibited strong statistical enrichment (Figure 15B).

Taken as a whole, these enrichment analyses suggest that the GA + CARB combination may be associated with multiple interconnected signaling pathways related to apoptosis, oxidative stress-associated responses, and cell cycle regulation. However, these pathway associations are derived from bioinformatic enrichment analyses and should be considered predictive and hypothesis-generating observations that require further biological and functional validation before definitive mechanistic conclusions can be drawn.

## 4. Discussion

The present study comprehensively investigated the biological effects of GA combined with CARB in cervical cancer cells using complementary two-dimensional and three-dimensional experimental models. Considering all experimental observations, the findings demonstrated that the GA + CARB combination was associated with enhanced cytotoxicity, increased apoptotic signaling, elevated oxidative stress, mitochondrial dysfunction, cell cycle dysregulation, and broad transcriptional alterations compared with either monotherapy alone. In contrast to our previous study evaluating a GA–cisplatin combination in HeLa cervical cancer cells, the present findings demonstrate that GA can also potentiate the biological activity of carboplatin, a clinically important platinum analog developed to improve tolerability relative to cisplatin [17]. Furthermore, the current study expands the mechanistic characterization of GA-based platinum combinations through integrated ROS/NAC rescue experiments and validation in three-dimensional tumor spheroid models, thereby providing additional translational relevance and biological support for the observed combination effects. Importantly, these treatment-associated effects were consistently more pronounced in HeLa cells than in HaCaT cells, suggesting a comparatively greater susceptibility of malignant cells to the combination regimen.

MTT-based viability analyses demonstrated dose- and time-dependent reductions in cellular viability following GA and CARB exposure in both cell lines, with HeLa cells exhibiting consistently lower IC_50_ values than HaCaT cells. CI analysis further indicated stronger synergistic interactions in HeLa cells relative to nontumoral keratinocytes. This differential response may be associated with the altered oxidative metabolism characteristic of cancer cells, which frequently operate under elevated basal ROS levels and exhibit reduced tolerance to additional oxidative burden [18]. Previous studies have shown that GA may display context-dependent antioxidant or pro-oxidant behavior depending on cellular redox balance and intracellular metal ion availability [10]. In the present study, the comparatively stronger response observed in HeLa cells may therefore reflect increased vulnerability of malignant cells to oxidative stress-associated injury rather than selective targeting in an absolute biological sense.

Flow cytometric apoptosis analysis demonstrated substantial increases in apoptotic cell populations following combined treatment. The GA + CARB regimen induced markedly higher total apoptotic fractions than either monotherapy alone while maintaining relatively limited necrotic cell accumulation. These findings suggest that treatment-associated cytotoxicity predominantly occurred through regulated apoptotic processes rather than extensive nonspecific membrane disruption. Apoptosis is widely recognized as a preferred mode of cancer cell elimination because it limits collateral tissue damage and inflammatory responses compared with uncontrolled cell death mechanisms [19]. From a translational perspective, apoptosis-dominant cellular responses may be advantageous because excessive necrotic death has been associated with inflammatory tissue injury and adverse treatment-related effects [20]. However, these observations remain restricted to controlled in vitro conditions and should not be directly extrapolated to therapeutic safety profiles without further in vivo validation.

ROS analyses further supported the involvement of oxidative stress-associated mechanisms in the observed treatment responses. Combination treatment markedly increased intracellular ROS levels, whereas NAC pretreatment substantially attenuated ROS accumulation and partially restored cellular viability. These findings indicate that oxidative stress is closely associated with the cytotoxic effects induced by GA + CARB exposure [21]. Nevertheless, NAC-based rescue experiments should be interpreted cautiously, as NAC may influence multiple redox-sensitive cellular pathways beyond direct ROS scavenging alone. Accordingly, the present findings support a contributory role for ROS-associated stress signaling rather than establishing oxidative stress as the exclusive mechanistic driver of apoptosis. Similar ROS-associated apoptotic responses following GA exposure have previously been described in lung and gastrointestinal cancer models [9,22].

ΔΨm analyses revealed substantial depolarization following combination treatment, consistent with disruption of mitochondrial integrity. The pronounced reduction in the JC-1 red/green fluorescence ratio observed in the combination group, together with increased *CYCS*, *CASP9*, and *CASP3* expression, supports the involvement of mitochondria-associated apoptotic signaling pathways [11]. Importantly, these mitochondrial alterations were accompanied by increased active caspase-3 immunoreactivity and elevated apoptotic fractions detected by Annexin V/PI staining. Although these observations collectively support activation of apoptosis-associated signaling cascades, the present study did not directly evaluate cytochrome c release kinetics, caspase cleavage dynamics, or upstream mitochondrial pore-forming events. Therefore, the proposed mechanistic interpretation should be considered biologically consistent but not definitively causal.

Cell cycle analyses demonstrated that the GA + CARB combination was associated with marked redistribution of HeLa cells toward the G2/M phase together with increased SubG1 accumulation. These findings were further supported by transcriptional alterations involving CDKN1A/p21, CDKN2A/p16, CDK4, and CDK6 [23]. CARB-mediated DNA damage has previously been linked to checkpoint activation and G2/M arrest in multiple tumor models [24], whereas polyphenolic compounds including GA have been associated with modulation of cyclin-dependent kinase signaling pathways [25,26]. The coordinated changes observed in the present study therefore suggest that combined treatment may interfere with cell cycle progression while simultaneously enhancing apoptosis-associated DNA fragmentation. However, because functional checkpoint assays were not performed, these observations should be interpreted as treatment-associated cell cycle redistribution rather than definitive proof of direct checkpoint inhibition.

Gene expression and immunocytochemical analyses demonstrated broadly concordant alterations in apoptosis- and cell cycle-related markers. Upregulation of *BAX*, *CASP3*, *CASP9*, *TP53*, *p21*, and *p16* together with the suppression of *BCL-2*, *CDK4*, and *CDK6* was accompanied by increased active caspase-3 immunoreactivity at the protein level. The marked elevation in the *BAX/BCL-2* ratio further supports increased susceptibility to mitochondrial apoptotic signaling [27,28]. Nevertheless, interpretation of these molecular findings should acknowledge that mRNA expression changes do not always directly correlate with protein activity or pathway functionality. Although immunocytochemical findings partially supported the transcriptional data, comprehensive protein-level validation using approaches such as Western blotting or phosphoproteomic analysis was not performed. Therefore, the observed transcriptional alterations should be considered preliminary molecular evidence and require further protein-based validation to more comprehensively confirm the proposed mechanism of action.

The three-dimensional spheroid experiments further strengthened the biological relevance of the observed findings. Compared with monotherapies, the GA + CARB combination induced greater spheroid disorganization, reduced spheroid diameter, diminished ATP-associated viability, and increased dead cell accumulation in live/dead fluorescence imaging. Because 3D spheroid systems better reproduce diffusion gradients, intercellular interactions, and structural heterogeneity than conventional monolayer cultures, these findings provide additional support for the biological activity of the combination under tumor-like conditions. However, spheroid systems still lack multiple components of the native tumor microenvironment, including vascularization, stromal signaling, and immune interactions. Consequently, the observed anti-spheroid effects should be interpreted as preclinical in vitro observations rather than direct indicators of therapeutic efficacy in vivo.

Bioinformatic analyses further suggested that GA- and CARB-associated targets are linked to multiple interconnected signaling pathways relevant to apoptosis regulation, oxidative stress responses, and cell cycle control. Hub gene analysis identified *TP53*, *AKT1*, *BCL2*, *CASP3*, *MYC*, and *EGFR* among the most interconnected nodes within the interaction network, whereas GO and KEGG enrichment analyses highlighted pathways related to apoptosis, ROS signaling, *PI3K/AKT* signaling, *p53* signaling, and cell cycle regulation [19,29,30]. These findings are biologically consistent with the experimental observations obtained in vitro. Nonetheless, bioinformatic enrichment analyses are inherently predictive and hypothesis-generating approaches that rely on existing database annotations and interaction probabilities. Therefore, these pathway associations require direct functional validation in future mechanistic studies.

Several limitations of the present study should be acknowledged. First, all experimental findings were obtained using in vitro cellular systems, which cannot fully reproduce the complexity of tumor biology in vivo. Second, although HaCaT cells were used as a comparative nontumoral model, immortalized keratinocytes do not fully represent normal cervical epithelial tissue. Therefore, the comparatively lower cytotoxicity observed in HaCaT cells should be interpreted cautiously as preliminary evidence of differential cellular sensitivity rather than definitive proof of treatment selectivity. Further validation using primary normal epithelial cells and in vivo models will be necessary to more accurately assess the selectivity profile of the GA + CARB combination. Third, mechanistic interpretations were primarily based on transcriptional profiling, flow cytometry, immunocytochemistry, and enrichment analyses; broader protein-level validation and pathway-specific functional inhibition studies were not performed. Consequently, the observed transcriptional alterations should be considered preliminary molecular evidence and warrant additional protein-based validation to more comprehensively confirm the proposed mechanism of action. Fourth, intracellular ROS measurements and NAC rescue experiments provide indirect evidence regarding oxidative stress-associated mechanisms but do not establish definitive causality. Additionally, although the Chou–Talalay method demonstrated a synergistic interaction between GA and CARB under the selected experimental conditions, synergy was evaluated using a single fixed-ratio concentration scheme. Future studies incorporating broader dose matrices and multiple combination ratios may provide a more comprehensive characterization of the interaction profile. Fifth, the study did not evaluate long-term clonogenic survival, migration/invasion behavior, CARB-resistant cell models, or in vivo pharmacodynamic responses. Consequently, conclusions regarding the relative superiority, mechanistic distinctions, or potential translational advantages of one platinum-based combination over the other cannot be drawn from the present findings and warrant dedicated comparative investigation in future studies. In addition, the relatively limited bioavailability of GA may restrict translational applicability unless optimized delivery systems such as nanoparticle-based or liposomal formulations are developed [31]. Finally, although the 3D spheroid experiments increased the physiological relevance of the study, these models still lack vascular, stromal, and immune microenvironmental components.

Overall, the present findings suggest that GA may enhance CARB-associated anticancer activity through mechanisms associated with oxidative stress responses, mitochondrial dysfunction, apoptosis-related signaling, and cell cycle dysregulation in cervical cancer cells. While the combination demonstrated stronger biological activity than either monotherapy alone across multiple experimental platforms, further in vivo, pharmacological, and translational investigations are required before potential clinical relevance can be determined.

## 5. Conclusions

The present study demonstrates that the GA + CARB combination exerts stronger anticancer activity in HeLa cervical cancer cells than either monotherapy alone. Combination treatment was associated with enhanced apoptosis, increased oxidative stress-related responses, mitochondrial membrane depolarization, cell cycle disruption, and broad transcriptional alterations involving apoptosis- and cell cycle-associated genes. These findings were further supported in 3D spheroid models, where combined treatment reduced spheroid growth, impaired spheroid integrity, and increased non-viable cell populations under tumor-like conditions.

In line with these findings, the integrated molecular, cellular, and bioinformatic findings suggest that GA may enhance CARB-associated anticancer responses through multiple interconnected biological pathways. However, because these findings were obtained under in vitro conditions, further in vivo and translational studies are required to determine the therapeutic relevance, pharmacological feasibility, and safety profile of this combination strategy in cervical cancer models.

## Figures and Tables

**Table 1:** Primer sequences used for RT-qPCR analysis. Primer sequences used for quantitative reverse transcription polymerase chain reaction (RT-qPCR) analysis of apoptosis-related genes (*BAX, BCL2, CASP3, CASP9, TP53,* and *CYCS*), cell cycle–related genes (*CDKN1A/p21, CDKN2A/p16, CDK4,* and *CDK6*), and housekeeping genes (*GAPDH* and *ACTB*).

Gene	Forward Primer (5′–3′)	Reverse Primer (5′–3′)
***BAX***	TTTGCTTCAGGGTTTCATCC	GACACTCGCTCAGCTTCTTG
***BCL2***	GGTGAACTGGGGGAGGATTG	GAGACAGCCAGGAGAAATCAAA
***CASP3***	TGGAACAAATGGACCTGTTGAC	AGGACTCAAATTCTGTTGCCAC
***CASP9***	CTTCGTTTCTGCGAACTAACAGG	GCACCACTGGGGTAAGGTTT
***TP53***	CAGCACATGACGGAGGTTGT	TCATCCAAATACTCCACACGC
***CYCS***	CCAATGATGGTGATGTTGAGAAAGG	TCTCCCCAGATGATGCCTTTG
***CDKN1A (p21)***	TGTCCGTCAGAACCCATGC	AAAGTCGAAGTTCCATCGCTC
***CDKN2A (p16)***	CGGCTGACTGGCTGGC	GGTCGGCGCAGTTGGG
***CDK4***	ATGGCTACCTCTCGATATGAGC	CATTGGGGACTCTCACACTCT
***CDK6***	TGGAGACCTTCGAGCACC	CACTCCAGGCTCTGGAACTT
***GAPDH***	GAAGGTGAAGGTCGGAGTC	GAAGATGGTGATGGGATTTC
***ACTB***	CATGTACGTTGCTATCCAGGC	CTCCTTAATGTCACGCACGAT

## Data Availability

The original contributions presented in this study are included in the article. Further inquiries can be directed at the corresponding authors.

## Abbreviations

ACTB	Beta-actin
AKT1	AKT Serine/Threonine Kinase 1
ANOVA	Analysis of Variance
ATP	Adenosine Triphosphate
BAX	BCL2 Associated X Protein
BCL2	B-cell Lymphoma 2
BP	Biological Process
CARB	Carboplatin
CASP3	Caspase-3
CASP9	Caspase-9
CCCP	Carbonyl Cyanide m-Chlorophenyl Hydrazone
cDNA	Complementary DNA
CDK4	Cyclin-Dependent Kinase 4
CDK6	Cyclin-Dependent Kinase 6
CDKN1A/p21	Cyclin-Dependent Kinase Inhibitor 1A
CDKN2A/p16	Cyclin-Dependent Kinase Inhibitor 2A
CI	Combination Index
CYCS	Cytochrome c, Somatic
DAB	3,3′-Diaminobenzidine
DCFH-DA	2′,7′-Dichlorodihydrofluorescein Diacetate
DMEM	Dulbecco’s Modified Eagle Medium
DMSO	Dimethyl Sulfoxide
DNA	Deoxyribonucleic Acid
FBS	Fetal Bovine Serum
FITC	Fluorescein Isothiocyanate
GA	Gallic Acid
GAPDH	Glyceraldehyde-3-Phosphate Dehydrogenase
GO	Gene Ontology
HPV	Human Papillomavirus
HRP	Horseradish Peroxidase
IC_50_	Half-Maximal Inhibitory Concentration
JC-1	5,5′,6,6′-Tetrachloro-1,1′,3,3′-tetraethylbenzimidazolocarbocyanine iodide
KEGG	Kyoto Encyclopedia of Genes and Genomes
MAPK	Mitogen-Activated Protein Kinase
MDM2	Mouse Double Minute 2 Homolog
MF	Molecular Function
MFI	Mean Fluorescence Intensity
MTT	3-(4,5-Dimethylthiazol-2-yl)-2,5-diphenyltetrazolium Bromide
NAC	N-Acetylcysteine
OMIM	Online Mendelian Inheritance in Man
PBS	Phosphate-Buffered Saline
PI	Propidium Iodide
PI3K	Phosphoinositide 3-Kinase
PPI	Protein–Protein Interaction
qPCR	Quantitative Polymerase Chain Reaction
ROS	Reactive Oxygen Species
RT-qPCR	Reverse Transcription Quantitative Polymerase Chain Reaction
SD	Standard Deviation
STRING	Search Tool for the Retrieval of Interacting Genes/Proteins
TP53	Tumor Protein p53
VEGFA	Vascular Endothelial Growth Factor A
ΔΨm	Mitochondrial Membrane Potential
